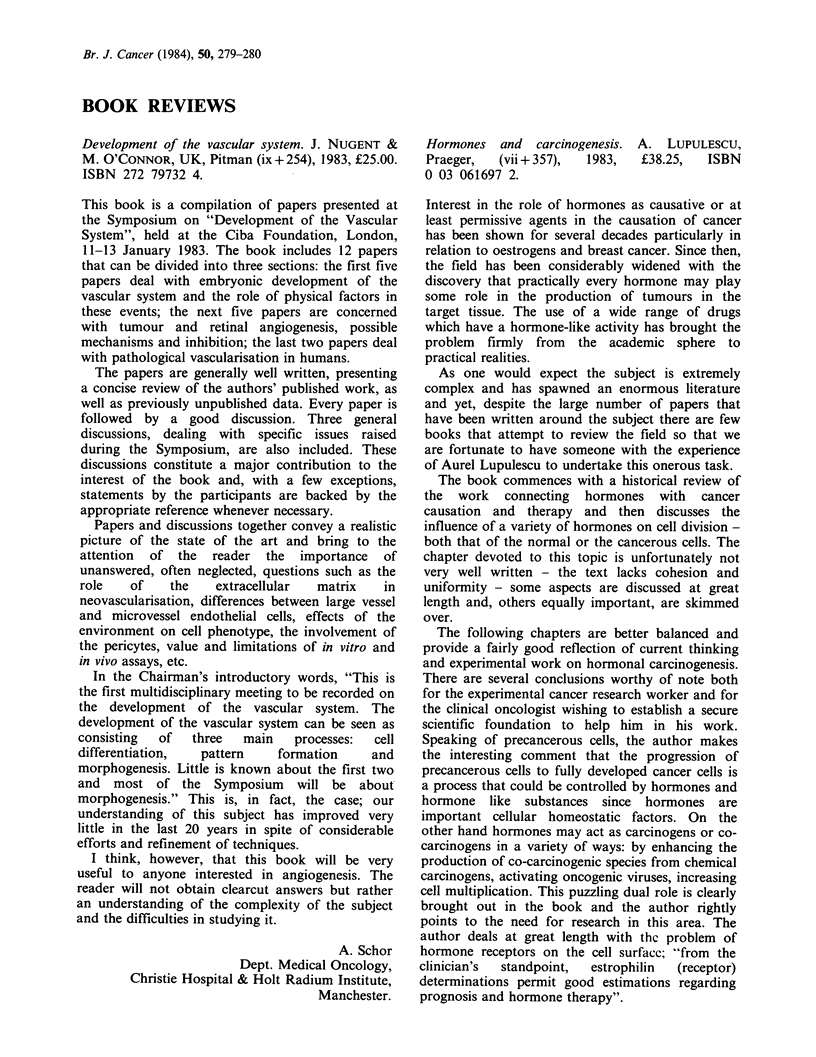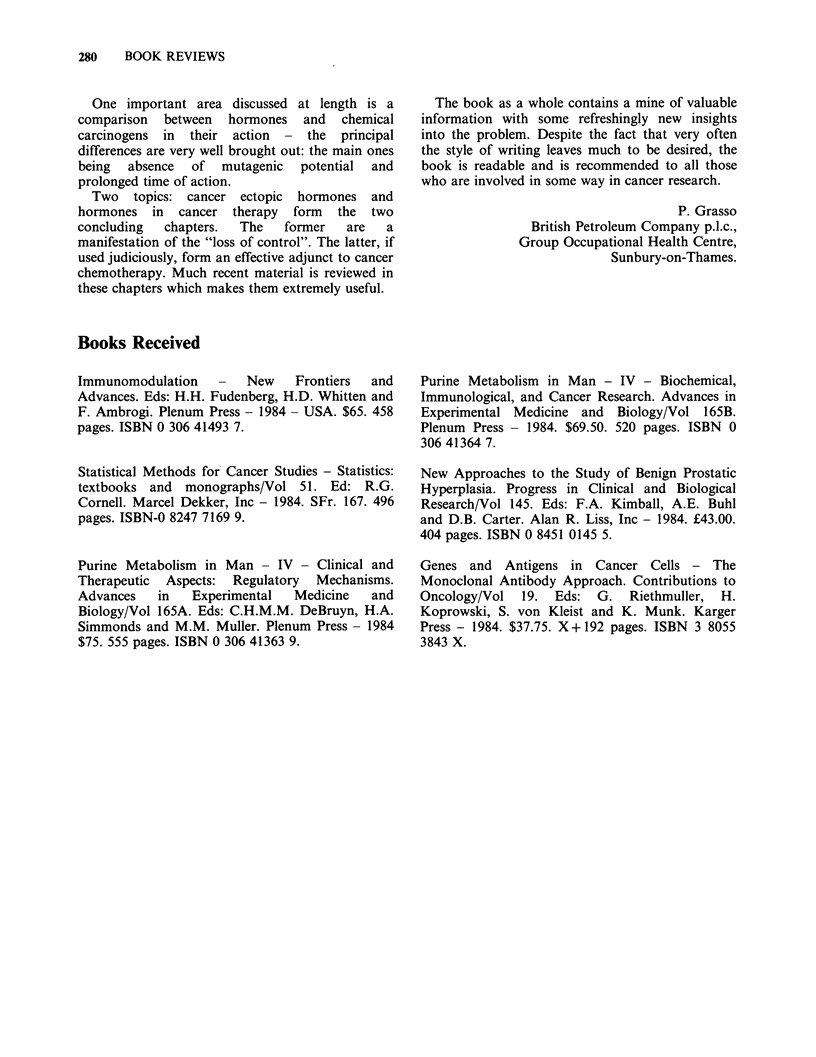# Hormones and carcinogenesis

**Published:** 1984-08

**Authors:** P. Grasso


*Hormones and carcinogenesis.* A. LUPULESCU, Praeger, (vii + 357), 1983, £38.25, ISBN 0 03 061697 2.

Interest in the role of hormones as causative or at least permissive agents in the causation of cancer has been shown for several decades particularly in relation to oestrogens and breast cancer. Since then, the field has been considerably widened with the discovery that practically every hormone may play some role in the production of tumours in the target tissue. The use of a wide range of drugs which have a hormone-like activity has brought the problem firmly from the academic sphere to practical realities.

As one would expect the subject is extremely complex and has spawned an enormous literature and yet, despite the large number of papers that have been written around the subject there are few books that attempt to review the field so that we are fortunate to have someone with the experience of Aurel Lupulescu to undertake this onerous task.

The book commences with a historical review of the work connecting hormones with cancer causation and therapy and then discusses the influence of a variety of hormones on cell division – both that of the normal or the cancerous cells. The chapter devoted to this topic is unfortunately not very well written – the text lacks cohesion and uniformity – some aspects are discussed at great length and, others equally important, are skimmed over.

The following chapters are better balanced and provide a fairly good reflection of current thinking and experimental work on hormonal carcinogenesis. There are several conclusions worthy of note both for the experimental cancer research worker and for the clinical oncologist wishing to establish a secure scientific foundation to help him in his work. Speaking of precancerous cells, the author makes the interesting comment that the progression of precancerous cells to fully developed cancer cells is a process that could be controlled by hormones and hormone like substances since hormones are important cellular homeostatic factors. On the other hand hormones may act as carcinogens or co-carcinogens in a variety of ways: by enhancing the production of co-carcinogenic species from chemical carcinogens, activating oncogenic viruses, increasing cell multiplication. This puzzling dual role is clearly brought out in the book and the author rightly points to the need for research in this area. The author deals at great length with the problem of hormone receptors on the cell surface; "from the clinician's standpoint, estrophilin (receptor) determinations permit good estimations regarding prognosis and hormone therapy".

One important area discussed at length is a comparison between hormones and chemical carcinogens in their action – the principal differences are very well brought out: the main ones being absence of mutagenic potential and prolonged time of action.

Two topics: cancer ectopic hormones and hormones in cancer therapy form the two concluding chapters. The former are a manifestation of the "loss of control". The latter, if used judiciously, form an effective adjunct to cancer chemotherapy. Much recent material is reviewed in these chapters which makes them extremely useful.

The book as a whole contains a mine of valuable information with some refreshingly new insights into the problem. Despite the fact that very often the style of writing leaves much to be desired, the book is readable and is recommended to all those who are involved in some way in cancer research.

P. Grasso
British Petroleum Company p.l.c.,
Group Occupational Health Centre,
Sunbury-on-Thames.